# Comparative Evaluation of the Serological Methods and the Molecular Genetics Techniques for the Diagnosis of *Encephalitozoon cuniculi* in Rabbits (*Oryctolagus cuniculus*)

**DOI:** 10.3390/microorganisms13071478

**Published:** 2025-06-25

**Authors:** Anca-Alexandra Doboși, Anamaria Ioana Paștiu, Sanda Andrei, Dana Liana Pusta

**Affiliations:** 1 Department of Genetics and Hereditary Diseases, Faculty of Veterinary Medicine, University of Agricultural Sciences and Veterinary Medicine Cluj-Napoca, 400372 Cluj-Napoca, Romaniadana.pusta@usamvcluj.ro (D.L.P.); 2 Department of Biochemistry, Faculty of Veterinary Medicine, University of Agricultural Sciences and Veterinary Medicine Cluj-Napoca, 400372 Cluj-Napoca, Romania; sandrei@usamvcluj.ro

**Keywords:** *Encephalitozoon cuniculi*, encephalitozoonosis, rabbit, serology, molecular genetics, diagnostic methods, Romania

## Abstract

*Encephalitozoon cuniculi* is a microsporidian widely spread in rabbits (*Oryctolagus cuniculus*) and other species, including humans, causative of neurological disorders or remaining in a latent state in the host organism. The aim of this study was to estimate the prevalence of *E. cuniculi* in rabbits located in the North-Western region of Romania, and to run a comparative diagnosis for *E. cuniculi* by multiple methods. A total of 381 rabbits were included, originating from households, family farms and wildlife, which were subjected to serological and/or molecular genetics diagnostic methods for *E. cuniculi* identification. Seropositivity of 43.02% (151/351) was obtained by ELISA, together with a 45.45% (110/242) prevalence from urine, feces and organs by nested PCR. Additionally, a prevalence of 48.39% (15/31) was identified by a comparative real-time PCR (qPCR). The urinary bladder is firstly reported for molecular *E. cuniculi* diagnosis, with a positivity of 30.56% (11/36) by nested PCR. Despite the disagreement between the diagnostic methods, the present results highlight the level of pathogen dissemination among rabbits in North-Western Romania that represents a risk for not only rabbits and other animals, but also for the general public by its zoonotic character.

## 1. Introduction

*Encephalitozoon cuniculi* is a microsporidial pathogen, described as a eukaryotic, unicellular, obligate intracellular and spore-forming protozoan parasite, but also being closely related to fungus [1,2]. At present, four strains (genotypes) have been identified. Strain I of this microorganism was firstly identified in rabbit (*Oryctolagus cuniculus*), this being its main host, and subsequently three other genotypes have been described as follows: genotype II—the murine strain, genotype III—the canine strain, and genotype IV—the human strain, though with no strict host specificity [3,4]. The zoonotic characteristic of *E. cuniculi* raises concerns, but infection usually occurs in immunocompromised humans, such as AIDS patients, organ transplant recipients, children, or older individuals [5].

In rabbits, transmission of *E. cuniculi* follows the horizontal route, mainly through oral ingestion of contaminated urine and feces, as well as the vertical route, through transplacental infection from doe to kit [2,6]. Host protection against the pathogen occurs through superior cell-mediated immunity, by a CD4^+^, CD8^+^ T lymphocyte and cytokine response, but also through humoral immunity, with the production of immunoglobulin M (IgM) and immunoglobulin G (IgG) antibodies [6,7]. The main targeted organs documented in the rabbit are the brain, kidney and eye lens, but heart or liver impairment have also been noted [8]. In the acute disease, rabbits show signs of cerebral impairment manifested by ataxia, head tilt, nystagmus, hemiparesis or paresis, tremors, and even epileptiform seizures with rolling, which can lead to death [8,9,10]. Renal symptoms tend to be hard to observe, but can include polydipsia, polyuria, anorexia and urinary incontinence [9], while the eye’s main lesion is usually phacoclastic uveitis [11]. Subclinical disease, characterized by an asymptomatic state, is the most common case of presentation [2], which is why diagnosis, treatment and prevention are key factors in managing *E. cuniculi* spread.

Diagnosis of *E. cuniculi* in rabbits consists of a number of methods with different sensitivity and specificity, mainly through ante- and postmortem techniques. Firstly, the presence of clinical signs, more exactly the neurological manifestations, is the indicator that puts encephalitozoonosis on the differential diagnosis list; however, the symptoms are not pathognomonic for the disease. Thereby, the main antemortem technique used is serological diagnosis, by identification of IgG and/or IgM antibodies through enzyme-linked immunosorbent assay (ELISA), indirect fluorescent antibody test (IFAT), carbon immunoassay (CIA), Western blot analysis and C-reactive protein (CRP) measurement [12]. Usually, it has been determined that the IgM titer ascends in an acute infection, the IgG titer elevates in a chronic or latent infection, while high titers of both IgM and IgG simultaneously can indicate an active infection (signalizing an acute infection, a reactivated infection or a reinfection) [13]. Postmortem methods of *E. cuniculi* identification are mainly based on molecular genetics techniques, respectively specific DNA detection using different polymerase chain reaction methods (PCR), such as conventional PCR, nested PCR or real-time PCR from urine, feces, cerebrospinal fluid (CSF) and organ tissues (brain, eye lens, kidney, liver, heart, lungs, spleen, intestines) [14,15,16]. Besides this, histopathological exam of the same tissues using different stains can help by identifying nonspecific inflammatory lesions, such as granulomatous meningoencephalitis and chronic interstitial nephritis, but more importantly by spore detection, that occurs more often in the brain and kidney [17]. A significant correlation has been found between results of serological and histopathological tests, while nested PCR also proved to be a good postmortem method of *E. cuniculi* diagnosis, with some limitations in regard to spore distribution in the examined tissue [14].

Therapeutic management of an acute encephalitozoonosis consists in a combination of anti-parasitic and supportive therapy, that aims to reduce spore-mediated inflammation and spore proliferation. Fenbendazole at a dosage of 20 mg/kg/day orally, for 28 days, has proved to be the most effective protocol against the pathogen; however, this does not guarantee a full recovery and its disappearance from the animal’s organism [6]. Along with this, steroidal or non-steroidal anti-inflammatories, systemic and topical ocular antibiotics, sedatives in case of seizures, antiemetics, fluid therapy and supplemental feeding are among the medications usually administered in an active infection [6,18]. Nevertheless, curing of the disease is most often not possible, which is why prophylaxis against *E. cuniculi* spread between animals and even humans is very important. Periodical serological testing in large rabbit populations with the elimination of positive individuals, preventive administration of fenbendazole, and thorough disinfection of the animal’s environment are some of the methods currently used in achieving this objective [6,18,19].

The aim of this study was to compare diagnostic methods of *E. cuniculi* on a population of rabbits, more exactly by serological evaluation using the ELISA technique and through molecular genetic analyses performed on excretions and tissue samples (nested PCR and qPCR). A second objective was to determine the prevalence of *E. cuniculi* for the studied rabbit population located in the North-Western region of Romania, as well as to evaluate its association with some environmental, clinical and animal factors.

## 2. Materials and Methods

### 2.1. Animals and Sampling

**Figure 1:**
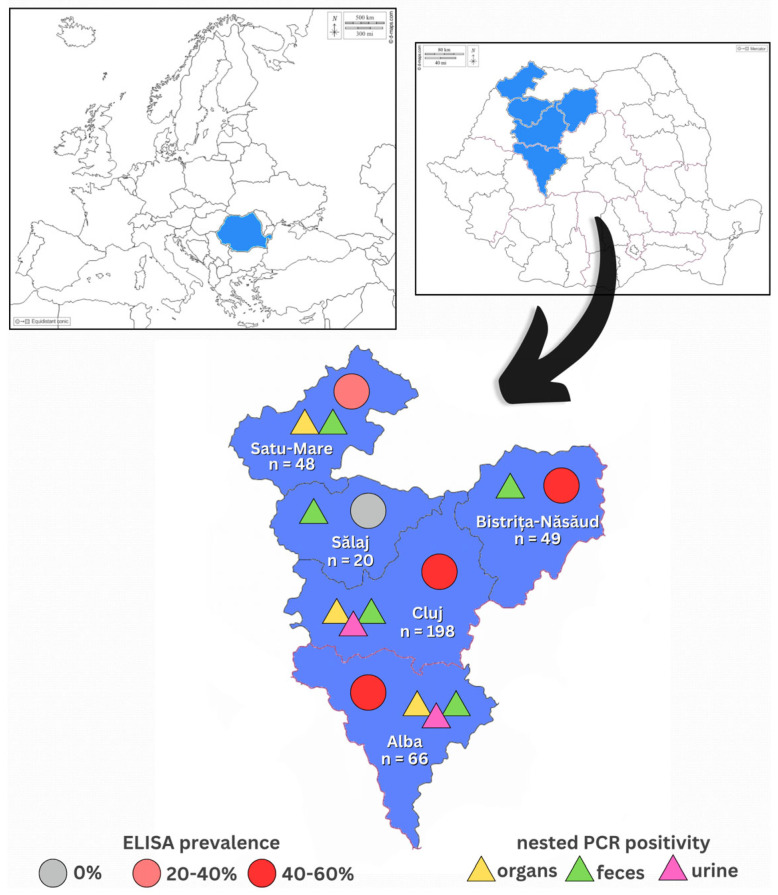
Map of the counties of North-Western Romania showing the results of *E. cuniculi* diagnosis. Each county shows the originating total number of rabbits included in this study. For each county, serological (ELISA) prevalence is marked with differently colored circles accordingly, while positive results of molecular genetics diagnosis (nested PCR) of the different samples tested are marked with differently colored triangles accordingly. Maps were taken from d-maps.com (https://d-maps.com/carte.php?num_car=2233, https://d-maps.com/carte.php?num_car=25495: accessed on 10 April 2025).

Between June 2022 and March 2025 a total number of 381 rabbits were examined and samples were collected for this study. The groups consisted of 66 pet rabbits, 312 farm rabbits and 3 European wild rabbits, all of which were the species *Oryctolagus cuniculus*. The pet rabbits were presented at the Clinic of New Companion Animals of the Faculty of Veterinary Medicine of Cluj-Napoca, Romania; the farm rabbits originated from the counties Alba, Bistrița-Năsăud, Cluj, Satu-Mare and Sălaj, Romania; the European wild rabbits came from the county Satu-Mare, Romania. The North-Western region of Romania was therefore analyzed, which is graphically represented in Figure 1. Clinical examination was performed and information regarding breed, sex, age, season of sampling, BCS (body condition score), vaccination status and health status were recorded for all but five individuals, from which organ tissues were directly received to be tested and the said information was incomplete.

The breeds of the domestic rabbits sampled were Californian, Continental Giant, Dwarf Rex, Flemish Giant, French Lop, Holland Lop, Hycole, Lionhead, Rex, Transylvania Giant, Vienna Blue and mixed breeds. For the age, two groups were established after the model of another study [20], where young animals were considered ≤4 months old and adult ones were >4 months old, of which 70 young and 311 adults. In regard to rabbit’s sex, 180 rabbits were male, 189 rabbits were female and 12 were of unidentified sex, either due to young age or missing data. Season of sampling was also recorded, where biological materials were collected from 65 rabbits in spring (March–May), 65 in summer (June–August), 157 in autumn (September–November) and 94 in the winter (December–February) season. Body weight of the animals was recorded to be between 500 g and 10 kg. For the BCS a scale from 1 to 5 was used, where 1 was interpreted as severe underweight, 3 as a normal weight and 5 as an obese rabbit [21]. In this study, the animals were categorized only in 2/5, 3/5 and 4/5 according to their weight and clinical aspect at the moment of examination. Vaccination status against RHD (rabbit hemorrhagic disease) and myxomatosis, for which rabbits usually require immunisation against, was also recorded, where 197 were vaccinated against RHD 1 and/or 2 and/or myxomatosis and the other 184 were completely unvaccinated or with an unknown vaccination status.

The rabbit’s clinical status was evaluated and labeled as symptomatic, for the 32 individuals that showed signs specific to encephalitozoonosis (neurological and/or renal), and asymptomatic, for the 337 individuals which were either clinically healthy or showed non-specific signs (gastrointestinal, dental, otic, respiratory, musculoskeletal or cutaneous diseases). For 12 rabbits, clinical status was unknown due to the fact that only cadavers were received, with no medical history provided. Among the specific clinical symptoms are numbered ataxia, head tilt, nystagmus, convulsive seizures with or without rolling, polydipsia, polyuria and urinary incontinence, while no ocular signs were observed in this study.

Blood, urine, feces and organ tissues (brain, eye lens, kidney, urinary bladder, liver, lungs, heart and spleen) were collected ante- and postmortem. The number of total samples collected from all 381 rabbits included in this study is as follows: 351 blood samples, 55 urine samples, 192 fecal samples and 287 tissue samples. All blood samples (n = 351) were destined for serological diagnosis, while the rest of materials (n = 534) were destined for diagnosis using molecular genetic techniques.

As antemortem sampling, blood, urine and feces were collected from 329 rabbits. Following their collection, all samples were individualized, refrigerated at 4–8 °C and transported to the laboratory on the same day. Blood (n = 322) was collected from each rabbit from the lateral saphenous vein with a 23G needle and a clot activator blood collection tube. Each sample was centrifuged at 1500 rpm for 10 min, sera were collected in 1.5 mL Eppendorf tubes. These were stored at −18 °C until batches of 44 samples were gathered for an ELISA plate to be analyzed (no longer than 4 weeks from sample collection). Urine samples (n = 35) were collected through spontaneous voiding of the bladder in sterile collection cups and prepared by centrifugation in 1.5 mL Eppendorf tubes at 1000 rpm for 10 min, followed by supernatant removal and storage of the cell pellet at −18 °C until further analysis. Fecal samples (n = 184) were collected in sterile plastic ziplock bags and were obtained by spontaneous defecation or from the rabbit’s enclosure and stored at −18 °C until testing. For urine and fecal samples, further DNA extraction for molecular diagnosis was performed in 48–72 h from the moment of collection.

For postmortem samples, all types of samples were collected at the moment of slaughter or during necropsy, from 52 rabbits. The 3 European wild rabbits were part of this group. Blood samples (n = 29) were collected in clot activator collection tubes from the jugular vein during slaughtering and further prepared with the same protocol as described before. Necropsy was performed and urine (n = 20) and fecal (n = 8) samples were collected from the urinary bladder and large intestine, respectively, following the same preparation protocol described before. In regards to organ samples, the following tissues were collected: brain (n = 52), eye lens (n = 51), kidney (n = 45), urinary bladder (n = 36), liver (n = 29), lungs (n = 28), heart (n = 26) and spleen (n = 20). Not all types of organs were available for collection from each rabbit, especially from those sent from the slaughterhouses. Each organ sample was appropriately labeled and DNA extraction was immediately performed (48–72 h).

Written consent of the animal owners for sample collection was obtained. This study was approved by the Animal Ethics and Welfare Committee of the University of Agricultural Sciences and Veterinary Medicine, Cluj-Napoca, Romania (No. 320/3 June 2022).

### 2.2. Serological Diagnosis

All sera samples were tested for anti-*E. cuniculi* antibody detection by using a commercial indirect enzyme-linked immunosorbent assay (ELISA, Medicago^®^, Uppsala, Sweden), following the manufacturer’s instructions. Prior to dispensing into the antigen-coated plates, serum samples were diluted 1:40 with phosphate buffer saline (PBS), positive and negative controls diluted 1:100 with PBS and the conjugate diluted 1:1000 with PBS. Each sera sample was introduced in an *E. cuniculi*-coated well and in a control antigen-coated well, thus being analyzed in replicates. Absorbances (A) were read at 450 nm. The results were interpreted using the manufacturer’s protocol (Sample A_450_ *E. cuniculi*-coated/Sample A_450_ control antigen-coated), where values > 2.0 were classified as positive for *E. cuniculi* antibodies and values ≤ 2.0 were classified as negative for *E. cuniculi* antibodies.

### 2.3. Molecular Genetics Diagnosis

#### 2.3.1. DNA Extraction

DNA extraction from biological samples was carried out with different extraction kits, depending on the type of tissue. All samples of urine were extracted with the Maxwell^®^ RSC Genomic DNA Kit (Promega, Madison, WI, USA), following the manufacturer’s instructions for “cell pellet” type samples, where the enzymatic digestion of the samples was performed with proteinase K at 56 °C. The Maxwell^®^ RSC Instrument (Promega, Madison, WI, USA) for automatic extractions was required for this process and the final products were stored at −18 °C until amplification.

For the fecal samples, the E.Z.N.A. Stool DNA Kit (Omega Bio-tek, Norcross, GA, USA) was used for the DNA extractions. Approximately 200 mg of samples were weighed and put in 2 mL Eppendorf tubes, enzymatic digestion being performed with proteinase K and glass beads at 70 °C for 24 h. The manufacturer’s protocol was followed, and the resulting DNA was kept at −18 °C until PCR testing.

The tissue samples were subjected to DNA extraction by using the DNeasy^®^ Blood & Tissue Kit (Qiagen, Hilden, Germany). Approximately 25 mg of each sample was sectioned into 1.5 mL Eppendorf tubes and enzymatic digestion with proteinase K was performed at 56 °C for 24 h, to assure an efficient degradation of the *E. cuniculi* spore wall. The manufacturer’s protocol was then followed, all products obtained being stored at −18 °C until further amplification.

#### 2.3.2. Nested PCR

Nested PCR was carried out as described previously by Katzwinkel-Wladarsch et al. [22]. This protocol was performed on all samples of urine, feces and tissues (n = 534). The positive control was represented by *E. cuniculi* spores, kindly provided by the Department of Pathobiology, University of Veterinary Medicine in Vienna, Austria. Ultrapure water was used as a negative control to ensure the reliability and accuracy of the PCR results.

#### 2.3.3. Real-Time PCR

For the Real-Time PCR (qPCR), the *Encephalitozoon cuniculi* Genesig Standard Kit (Primerdesign Ltd.^TM^, Hampshire, UK) was used, following the manufacturer’s instructions. The reaction mixture total volume of 20 µL consisted of 10 µL Master Mix (GoTaq^®^ DNA Polymerase, Promega, Madison, WI, USA), 1 µL specific *E. cuniculi* primer provided by the kit, 4 µL nuclease-free water and 5 µL DNA. The Azure Cielo^TM^ Real-Time PCR System (Azure Biosystems, Dublin, CA, USA) was used for the following incubation protocol: 95 °C for 2 min, followed by 40 cycles of 95 °C for 15 s and 60 °C for 1 min. This protocol was performed on a total of 122 samples from 31 rabbits, respectively 102 organ tissue samples, 10 urine samples and 10 fecal samples.

#### 2.3.4. Gel Agarose Electrophoresis

For the nested PCR amplifications, results were read by a gel agarose electrophoresis 1.5% with RedSafe™ (iNtRON Biotechnology, Boston, MA, USA) solution under UV transilluminator. Positive and negative control samples were run in each PCR reaction.

### 2.4. Statistical Analysis

The serological status for *E. cuniculi* (positive/negative), as well as the specific *E. cuniculi* DNA identification from each type of biological sample (positive/negative) were taken as dependent variables in relation to the animal’s data (age, sex, season of sampling, rearing system, BCS, vaccination status, health status and county of origin) by analysis using Pearson’s chi-squared test for independence. 95% Confidence Intervals (95% CI) for each of the variables were calculated using EpiTools 0.5–10.1 (Sergeant, ESG, 2018) (http://epitools.ausvet.com.au, accessed on 10 April 2025) and statistical significance was set at *p* < 0.05.

For the correlation of diagnostic methods, Cohen’s kappa index (*κ*) was calculated. The sensitivity (Se), specificity (Sp), positive and negative predictive values (PPV and NPV) of the ELISA and qPCR were calculated using nested PCR as golden standard method. The result (positive/negative) of each sample (blood, urine, feces and tissues) were analyzed individually and then also compared. A rabbit was considered positive by a given method if at least one sample tested positive using that method (qPCR or nested PCR).

A multivariate logistic regression analysis was conducted to evaluate the association between potential risk factors and the outcomes of interest. The analysis was applied to 242 rabbits for nested PCR and 351 rabbits for ELISA. Two separate models were developed, using ELISA and nPCR test results as dependent variables, respectively. Independent variables included sex, age, vaccination status, season of sampling, clinical status and rearing system. Odds ratios (ORs) and 95% CI were calculated for each predictor. A *p*-value < 0.05 was considered statistically significant. The following categories were set as reference in the model: Age: ≤4 months, Sex: Male, Season: Spring, Rearing system: Pet, Vaccination status: Unvaccinated, Clinical status: Asymptomatic. All other categories were compared against these references.

Statistical analysis was performed using the EpiInfo 2000 software (CDC, Atlanta, GA, USA) and Win Episcope 2.0 software (Zaragoza, Spain).

## 3. Results

### 3.1. Serological Diagnosis

A total seroprevalence (anti-IgG antibodies) of 43.02% (151/351, 95% CI: 37.94–48.25) was determined, with statistically significant differences depending on age, season of sampling, BCS, clinical status and county of origin, which are shown in Table 1.

### 3.2. Molecular Genetics Diagnosis

From the total number of 242 rabbits tested by nested PCR from either urine, feces or organs, a positivity of 45.45% (110/242, 95% CI: 39.30–51.75) was identified.

For the nested PCR from urine samples, the prevalence of 27.27% (15/55, 95% CI: 17.28–40.23) was identified, with significant differences depending on the rearing system and BCS, which can be observed in Table 2.

Nested PCR from fecal samples revealed a total prevalence of 37.50% (72/192, 95% CI: 30.96–44.53), with significant differences depending on age, sex, season of sampling, rearing system, BCS, clinical status and county of origin, which are shown in Table 3.

Organ samples analyzed by nested PCR demonstrated a prevalence of 61.54% (32/52, 95% CI: 47.02–74.70), where results depending on the rabbit’s sex, season of sampling, rearing system, clinical status and county of origin were statistically significant, as observed in Table 4.

In regards to the different tissues tested by nested PCR, a positivity of 21.95% (63/287, 95% CI: 17.55–27.09) was obtained, with no relevant difference between results, as seen in Table 5.

Additionally, qPCR was performed on urine, feces or organs from 31 rabbits, where the total prevalence obtained was 48.39% (15/31, 95% CI: 31.97–65.16). From the total of 102 tissue samples tested, the prevalence of *E. cuniculi* DNA was 29.41% (30/102, 95% CI: 21.45–38.87).

### 3.3. Comparison of Diagnostic Methods

#### 3.3.1. Comparison of ELISA and Nested PCR

In this study 212 rabbits were tested using both ELISA and nested PCR, results shown in Table 6. The seroprevalence of *E. cuniculi* antibodies was 58.96% (125/212, 95% CI: 52.24–65.37), while the prevalence of *E. cuniculi* DNA was 43.3% (92/212, 95% CI: 36.90–50.13). A total of 54 rabbits tested positive by both ELISA and nested PCR, while 49 rabbits were negative by both methods. However, 71 rabbits tested positive by ELISA but negative by nested PCR, and 38 rabbits tested negative by ELISA but positive by nested PCR.

#### 3.3.2. Comparison of qPCR and Nested PCR

A total of 31 rabbits were tested using both qPCR and nested PCR, which can be seen in Table 7. The prevalence of *E. cuniculi* DNA by qPCR was 48.39% (15/31, 95% CI: 31.97–65.16), while the prevalence of *E. cuniculi* DNA by nested PCR was 54.84% (17/31, 95% CI: 37.77–70.84). A total of 10 rabbits tested positive by both qPCR and nested PCR, while 9 rabbits were negative by both methods. However, 5 rabbits tested positive by qPCR but negative by nested PCR, and 7 rabbits tested negative by qPCR but positive by nested PCR.

#### 3.3.3. Diagnostic Performance Indicators

Nested PCR was selected as the reference (gold standard) diagnostic method, due to its ability to directly detect *E. cuniculi* DNA and its higher sensitivity reported in previous studies [14]. This method served as the reference for evaluating the performance of ELISA and qPCR. Diagnostic performance indicators, including Se, Sp, PPV, NPV, accuracy and estimated infection prevalence were calculated based on these comparisons. These results are summarized in Table 8.

#### 3.3.4. Multivariate Logistic Regression

The results of the multivariable logistic regression analysis identified several significant predictors for ELISA and nested PCR positivity. Detailed odds ratios (ORs), 95% CI, and *p*-values for each variable are presented in Table 9.

#### 3.3.5. Overview of All Diagnostic Methods Applied

Comparative results of all diagnostic tests performed in 6 rabbits and their symptomatology are observed in Table 10. While one single individual had positive results for *E. cuniculi* in all tests performed, the other 5 rabbits showed positivity in either serological and/or molecular genetics methods, among these being an asymptomatic rabbit as well.

## 4. Discussion

Over the last decades, different diagnostic tools have been used in order to identify *E. cuniculi* infection in rabbits, not just in the individuals manifesting specific clinical signs, but also as a screening method for general populations that could be asymptomatic carriers and should be removed from groups and reproduction. So far, the serological technique proved good results especially on large rabbit populations, as it can be performed antemortem and relatively easy, but molecular genetics methods also showed relevance in the efficient microsporidial identification, mostly on animal tissues [6]. Even if not all diagnostic techniques are suitable for antemortem diagnosis, this study aimed to evaluate and compare the different methods available, both ante- and postmortem diagnostics. This way, efficiency of the techniques could be evaluated, while also having a glance over the prevalence of *E. cuniculi* among rabbits in the North-Western region of Romania.

### 4.1. Serological Diagnosis

Identification of anti-*E. cuniculi* antibodies in rabbits represents a well-used method of antemortem diagnosis; however, its important limitation remains to be the uncertainty as a confirmative diagnosis [6]. This is explained by the fact that the presence of antibodies simply confirms an exposure to the microsporidian, but without information to the exact moment of contact, and only multiple negative results can exclude the infection [2,6]. In this study, the ELISA technique chosen allowed for a qualitative antibody detection, also supported by other research [23], and with good feasibility for a large sample load and as a screening method.

The total prevalence identified by ELISA testing in this study was of 43.02% (151/351, 95% CI: 37.94–48.25), which is quite a concerning percentage considering that 132 of the positive rabbits were asymptomatic, therefore latent carriers of *E. cuniculi*. Comparable prevalences were reported all around the world, with higher numbers of 59.56%, 67.2% and 70.5% in Italy, respectively [24,25,26], 52% and 59% in U.K., respectively [8,27], 69.7% in Austria [14], 67.8% in Taiwan [28], 62% in the USA [29] and 81.7% in Brazil [9]; lower prevalences were found also in Italy—31.6% [20], in Germany—39.45% [30], in Finland—29.2% [31] and in Korea—22.6% [32]. In the mentioned studies, ELISA was the predominant serological method used, which proves its large applicability, especially in large populations.

Seropositivity in relation to rabbit’s age, season of sampling, clinical status, BCS and county of origin showed statistical significance. The relevantly (*p* = 0.0001) higher seropositivity of 48.23% (136/282) in the adult group than 21.74% (15/69) in the young group is in accordance with other studies, which report that young rabbits between 4 and 8 weeks of age experience a decline in maternal antibodies, potentially leading to seronegative results [20,23,24]. The rabbit’s seroprevalence by the season of sampling showed the bigger percentages in spring and autumn, 65.57% (40/61) and 45.27% (67/148) respectively, which proved a significant difference (*p* = 0.0001) contrary to another study [33]. These results can be supported by a study on *Enterocytozoon bieneusi* in cattle, based on the fact that microsporidian spores can have a better resistance and transmission in spring and autumn seasons that have favorable temperature and rainfall conditions, compared to the other seasons with extreme temperatures [34]. Significance (*p* = 0.049) was shown by clinical status, 61.29% (19/31) of symptomatic animals showing seropositivity compared to 41.25% (132/320) of the asymptomatic group, which is also supported by other reports [25,35], considering that *E. cuniculi* presents a high possibility of being the causative agent of disease in case of rabbits with neurological symptoms. Another interesting finding was the significantly (*p* = 0.021) higher seroprevalence for rabbits with a BCS of 2/5 and 4/5, respectively 55.56% (5/9) and 45.24% (19/42), that could indicate a possibly higher immunosuppression for the individuals with a poorer physical condition. Lastly, rabbit’s county of origin did reveal significant differences, Alba, Cluj and Bistrița-Năsăud having closely high seropositivity, 51.52% (34/66), 47.95% (82/171) and 46.94% (23/49) respectively, while Satu-Mare and Sălaj counties presented a lower prevalence of 26.67% (12/45) and even 0% (0/20) respectively. The groups tested were, however, not homogenous and Cluj also contained most of the symptomatic animals, therefore further studies on larger populations from each county and also regions of Romania are needed.

### 4.2. Molecular Genetics Diagnosis

The *E. cuniculi* DNA identification from various biological materials has become one of the methods more used and attempted recently, both ante- and postmortem, with a greater certainty in regards to a confirmative diagnosis. Up to now, nested PCR and qPCR seem to be the best methods for *E. cuniculi* identification, nested PCR being described by the thorough DNA amplification through two successive PCR reactions, using the product of the first reaction in the second one [14], while qPCR appears to present a higher sensibility for DNA detection, increased throughput and reduced risk of PCR amplicon carry-over [36]. These proved to work well with organs, such as brain, eye lens, kidney, liver, lungs, heart, spleen and intestines [14,16], and to a lesser extent with urine and feces, where short and intermittent spore shedding intervenes [15]. Conventional PCR proved good results mainly for eye lens, especially with lesions of phacoclastic uveitis, where the spore load is usually large enough to prove positivity [14,37]. In the absence of such eye lesions in our study, conventional PCR was therefore not performed, the other methods with higher sensitivity being chosen for the present samples.

Nested PCR of urine samples showed a total prevalence of 27.27% (15/55, 95% CI: 17.28–40.23), which is a relatively low percentage despite the small sample size. Also using this method, other countries reported quite low prevalences as well, such as 29.7% and 39.5% in Austria, respectively [14,38], 48.7% in Germany [39], 7.78% in Japan [15] and 32% in Turkey [40], while conventional PCR in Egypt showed the lowest percentage of 2.85% [41], which only further proves that this biological material is not the most reliable in identifying the microsporidian, if the moment of spore shedding is not caught. The rearing system showed significance (*p* = 0.0001) in relation to molecular urine positivity, a higher prevalence of 35.48% (11/31) in pet rabbits compared to 16.67% (4/24) in family farms being obtained. This could be associated with the pet category having most symptomatic rabbits of this study that could have been caught in the phase of spore shedding; however, clinical status did not prove to be of relevance, also supported by another study [14]. BCS was the only other variable with relevance (*p* = 0.0060) related to urine positivity, more exactly the bigger percentages, again, for the 2/5 and 4/5 scores, with 50% (3/6) and 37.50% (3/8) respectively, supporting the same theory of infection susceptibility for unbalanced organisms’ with lower immunity. Ultimately, the sporadic shedding of *E. cuniculi* spores has been confirmed multiple times, and has been reported to decline and end at about 98 days post-infection [6], which makes for an unreliable sample type for this diagnosis.

A total prevalence of 37.50% (72/192, 95% CI: 30.96–44.53) was determined by nested PCR from fecal samples, which shows a relatively reduced positivity. Even lower percentages were obtained by the same technique, 5.6% in Japan [15] and 5.8% in China [42] respectively, while conventional PCR in Brazil revealed a 2.56% [43] positivity. Even if all variables except the vaccination status were revealed to be of significance, we considered only age, season of fecal sampling, rearing system, clinical status and county of origin to be discussed. Interestingly, the younger category proved to have a relevantly (*p* = 0.0031) higher prevalence of 58.54% (24/41) than the adult rabbits with 31.79% (48/151), which is in disagreement with other results [42]. This could be explained by a potentially higher dissemination of *E. cuniculi* spores by the oral route in younger rabbits housed with siblings and parents, followed by fecal elimination. Season of fecal sampling proved significance (*p* = 0.0002) by the highest molecular positivity of 65% (26/40) in summer, followed by 37.33% (28/75) in autumn, which could suggest better spore resistance of this sample type in warmer temperatures. Cleaner environmental conditions for pet rabbits is supported by the significantly (*p* = 0.0001) lower prevalence of 5% (2/40) *E. cuniculi* feces DNA identification, compared to farm rabbits with 46.05% (70/152). Another striking discovery was the far higher fecal positivity of 41.18% (70/170) in the asymptomatic group than 9.09% (2/22) for symptomatic animals, also of significance (*p* = 0.0071), which only raises awareness on *E. cuniculi*’s ability of going unnoticed, as well as the importance of environment disinfection. At last, statistical relevance was proved (*p* = 0.0001) also by rabbits’ county of origin, where some interesting results for fecal samples appeared. While in Sălaj county the obtained prevalence of 13.33% (2/15) was the lowest, this sample type was the only one to show positivity here, demonstrating the diagnosis variability. Besides this, the highest prevalence of 82.14% (23/28) was found in Satu-Mare county, which is in disagreement with the far lower seroprevalence (26.67%) and urine positivity (0%), showing another disparity between the samples and the different diagnosis methods performed. In spite of the many analytical significances, the scarce number of studies performed on fecal *E. cuniculi* DNA identification so far continues to support the short and intermittent excretion of spores through feces, as well as the general reduced prevalence in this sample type [15,42], that does not make it the most appropriate for a confirmative diagnosis in absence of a clinical manifestation.

Molecular positivity for *E. cuniculi* determined from organs using nested PCR was 61.54% (32/52, 95% CI: 47.02–74.70), which is quite an alarming number considering the majority of rabbits included were asymptomatic. By the same technique, comparative results of 63.6% and 42.1% organ positivity in Austria [14] and 59.6% brain positivity in Iran [12] were reported, while conventional PCR generally reports a lower organ prevalence of 25.4% in Austria [14], 36.4% in Italy [44] and even a maximum 100% positivity in Egypt [11]. The most sensible tissue for conventional PCR proved to be the ocular globe, more exactly the eye lens, supported by studies from Austria [14], Turkey [37] and UK [45], with prevalence of 100%, 63% and 33.3% respectively. Overall, nested PCR is clearly considered to be of a higher sensitivity than conventional PCR for organ testing; however, the spore distribution in tissues remains a limitation.

Asymptomatic rabbits demonstrated 63.89% (23/36) positivity in organs, compared to 60% (3/5) and 54.55% (6/11) in symptomatic and individuals with unknown clinical status respectively. Despite the close percentages, its relevance (*p* = 0.0001) highlights the level of *E. cuniculi* spread among the general population. Another finding was in relation to the rabbits’ rearing system, where 68.75% (22/32), 52.94% (9/17) and 33.33% (1/3) organ positivity for farm, pet and wild rabbits respectively, was identified. With statistical relevance (*p* = 0.0002), it is important to raise the question of rabbit meat consumption risks, since *E. cuniculi* owns a zoonotic character and most farm rabbits are destined for this, but only one study on mice proved that fermented pork meat infected could represent a potential source of transmission to humans [46]. Despite the low number of European Wild rabbits (*O. cuniculus*) taken into this study (n = 3), nested PCR of organs showed positivity for one individual, a 4.4% positivity also being identified from the brain and kidney of the same rabbit species by both nested and real-time PCR in Germany [47] and a 9.72% positivity from brain, kidney and skeletal muscle of Eastern Cottontail rabbits (*Sylvilagus floridanus*) by conventional PCR in Italy [48]. The wildlife system as an *E. cuniculi* reservoir is a study direction to further be analyzed. The highest tissue positivity of 72.73% (16/22) in the winter season and lowest of 0% (0/4) in summer could only suggest a weakened rabbit immune system in the cold season, translated through an increased spore proliferation and organism infection during this time of year. The county of Cluj showed the best organ positivity of 63.64% (28/44), but this group also contained most symptomatic rabbits, which does not make for a definitive conclusion based on the area of origin. Larger rabbit populations in both symptomatic and asymptomatic antemortem states should be further tested to establish a clearer conclusion, but these results need to be taken into consideration and meat consumption needs to be better controlled to limit human exposure.

In most studies, *E. cuniculi* DNA identification is performed on the target organs of *E. cuniculi*, more exactly the brain, kidney and ocular globe [11,37,44,45], but others like liver, lungs, heart, spleen and intestine have also been reported for diagnosis [14,16]. Focusing on the predilection organs, our research reports the highest prevalence in the kidney (26.67%), followed by brain (21.15%) and eye lens (13.73%), consistent with results from other studies, which also proved the brain and kidney as the most affected organs, also sustained by histopathological diagnosis [14,16]. The novelty of urinary bladder testing conducted in our study revealed promising results, with a 30.56% (11/36) prevalence, even higher than the previously mentioned tissues. Even if residual urine that contained infective spores could be the reason why specific DNA was identified in the bladder, this finding is substantial and should be further investigated.

### 4.3. Comparison of Diagnostic Methods

The most important comparison to be taken into consideration is between serological and molecular genetics methods by nested PCR, which was performed on a total of 212 rabbits. Overall, a higher seroprevalence was identified in comparison to nested PCR positivity. This can be explained by the molecular diagnostic sample types’ main limitations, represented by the intermittent spore shedding through excretions and the uneven or absent spore load caught in the organ sections [1,6], that could lead to false negative results. In relation to rabbit’s age, seroprevalence turned out higher in the adult group (69.57%) and molecular positivity higher for the young ones (60.78%), which could be explained by the latter having seronegative results due to the disappearance of maternal antibodies, but concurrent possible *E. cuniculi* infection translated through spore DNA identification. Moreover, symptomatic rabbits had a relatively high (70.83%) seroprevalence, while the asymptomatic group obtained the bigger percentage (44.68%) for nested PCR, which only further supports *E. cuniculi*’s latency and the discrepancy in clinical signs display.

The diagnostic agreement between ELISA and nested PCR for *E. cuniculi* was assessed using Cohen’s Kappa statistic. The resulting Kappa value indicated no agreement beyond chance, a finding further supported by a non-significant *p*-value (*p* = 0.94). These results suggest that the two methods may detect different aspects or stages of infection—ELISA identifying past exposure through antibodies, and nested PCR detecting current infection via parasite DNA. Although limited comparative data exist, our study estimated the sensitivity and specificity of ELISA to be 58.7% and 40.83%, respectively, when using nested PCR as the reference standard. While these values are moderate, they support the potential applicability of ELISA as a supplementary diagnostic tool. Nevertheless, nested PCR remains the gold standard for the accurate identification of *E. cuniculi* DNA and should be included in routine diagnostic protocols for encephalitozoonosis [14].

Multivariate logistic regression analysis identified age and season as significant predictors of a positive ELISA result. All ORs are interpreted relative to the reference categories: Age: ≤ 4 months, Sex: male, Season: spring, Rearing system: pet, Vaccination status: unvaccinated, Clinical status: asymptomatic. Adult rabbits showed a 4.5-fold higher odds of testing positive compared to juveniles, indicating a strong association between age and cumulative exposure (OR = 4.50, 95% CI: 2.19–9.23, *p* < 0.0001). This finding suggests cumulative exposure to *E. cuniculi* over time, which is consistent with the chronic nature of the infection. Season of sampling also influenced ELISA results. Specifically, rabbits sampled in winter had significantly lower odds of seropositivity compared to those sampled in spring (OR = 0.45, 95% CI: 0.26–0.79, *p* = 0.003), suggesting a possible seasonal pattern in transmission or seroconversion. These findings highlight the influence of both biological and environmental factors on disease dynamics.

For nested PCR positivity, seasonal variation was also observed, with samples collected in summer being more likely to test positive compared to those collected in spring (OR = 2.72, 95% CI: 1.39–5.44, *p* = 0.003). This may reflect a higher rate of active infection or shedding during warmer periods, possibly due to stress or environmental persistence of spores. However, other factors, such as sex, vaccination status, rearing system, and clinical signs, did not show statistically significant associations with either ELISA or PCR outcomes. Interestingly, clinical status was not significantly associated with either seropositivity (OR = 1.67, *p* = 0.329) or nested PCR positivity (OR = 0.77, *p* = 0.628), reinforcing the idea that subclinical infections are common in *E. cuniculi*–infected rabbits.

By comparing the molecular genetics techniques and their results, the total prevalence for both nested PCR and qPCR for the 31 rabbits analyzed were close, 54.84% (17/31) and 48.39% (15/31) respectively, and no relevant differences observed between the grouped variables. Even so, nested PCR seemed to have a slightly higher positivity, which further sustains our choice of this diagnostic method as the golden standard. More studies comparing these two molecular techniques in the *E. cuniculi* diagnosis are required for a clearer conclusion. Taking nested PCR as the golden standard, sensitivity and specificity of ELISA and qPCR were assessed. While the sensitivity had an almost identical result, 58.7 and 58.8 respectively, specificity was rather different, 40.83% for ELISA and 64.3% for qPCR.

By analyzing the sole 6 rabbits which had all diagnostic methods performed, a general variability of positivities, regardless of the sample type or method, could be noticed. Interestingly enough, qPCR from organs turned out to be the only positive diagnosis for all individuals; however, no studies to support a higher accuracy for this diagnosis above others exist yet.

Although the results of the present study are substantial and provide a valuable foundation for future research on rabbit populations in Romania, several limitations were identified. These include the uneven distribution of the rabbit groups sampled and the fact that not all types of biological samples could be collected from each individual. Other limitations include the inability of serological testing to determine the timing of exposure to *E. cuniculi*, as well as the use of a qualitative ELISA assay that detects only IgG antibodies and does not allow for the quantitative assessment of either IgM or IgG. Additionally, it was not possible to apply qPCR to all the samples that were tested using nested PCR. Furthermore, the short and intermittent shedding of *E. cuniculi* spores in urine and feces may have affected detection by molecular methods, while the low, absent, or uneven distribution of spores in organ sections could have impacted molecular analysis outcomes.

## 5. Conclusions

This study confirms the presence of *Encephalitozoon cuniculi* in rabbits from counties in North-Western Romania. While serological testing using ELISA allowed for large-scale screening, molecular techniques such as nested PCR provided more definitive diagnostic results, supporting their role in confirmatory testing. Significant discrepancies were observed between ELISA and nested PCR results in paired samples, and notably, the urinary bladder was identified for the first time as a potential site for harboring microsporidian spores.

The implications for public health, particularly concerning human exposure through rabbit cohabitation and meat consumption, warrant further investigation. However, the priority remains the improvement and standardization of *E. cuniculi* diagnostic methods in animal populations to effectively monitor and reduce the risk of transmission.

## Figures and Tables

**Table 1:** *E. cuniculi* prevalence based on ELISA testing performed in 351 rabbits from the North-Western region of Romania.

Rabbit Data	No. Rabbits Tested	No. Positive Rabbits	Prevalence (%)	CI 95%	*p* *
Age					0.0001
Young (≤4 months)	69	15	21.74	13.64–32.82	
Adults (>4 months)	282	136	48.23	42.46–54.04	
Sex					0.273
Male	165	65	39.39	32.26–47.01	
Female	179	84	46.93	39.76–54.23	
Unidentified	7	2	28.57	8.22–64.11	
Season of sampling					0.0001
spring	61	40	65.57	53.05–76.25	
summer	62	22	35.48	24.74–47.92	
autumn	148	67	45.27	37.47–53.31	
winter	80	22	27.50	18.92–38.14	
Rearing system					0.066
Pet	48	27	56.25	42.28–69.30	
Family farm	303	124	40.92	35.53–46.54	
BCS					0.021
2/5	9	5	55.56	26.67–81.12	
3/5	300	127	42.33	36.87–47.99	
4/5	42	19	45.24	31.22–60.05	
Vaccination status					0.743
vaccinated	186	78	41.94	35.08–49.12	
unvaccinated	165	73	44.24	36.88–51.87	
Clinical status					0.05
symptomatic	31	19	61.29	43.82–76.27	
asymptomatic	320	132	41.25	35.99–46.72	
County of origin					0.0001
Alba	66	34	51.52	39.71–63.15	
Bistrița-Năsăud	49	23	46.94	33.70–60.62	
Cluj	171	82	47.95	40.59–55.40	
Satu-Mare	45	12	26.67	15.96–41.04	
Sălaj	20	0	0	0–16.11	
**Total**	**351**	**151**	**43.02**	**37.94–48.25**	

* Chi-square test; BCS—body condition score; CI—confidence interval.

**Table 2:** *E. cuniculi* prevalence based on nested PCR from urine samples performed in 55 rabbits from the North-Western region of Romania.

Rabbit Data	No. Rabbits Tested	No. Positive Rabbits	Prevalence (%)	CI 95%	*p* *
Age					0.110
Young (≤4 months)	9	0	0	0–29.91	
Adults (>4 months)	46	15	32.61	20.87–47.03	
Sex					0.124
Male	23	4	17.39	6.98–37.14	
Female	32	11	34.38	20.41–51.69	
Season of sampling					0.785
spring	6	2	33.33	9.68–70.00	
summer	12	2	16.67	4.70–44.80	
autumn	15	5	33.33	15.18–58.29	
winter	22	6	27.27	13.15–48.15	
Rearing system					0.0001
Pet	31	11	35.48	21.12–53.05	
Family farm	24	4	16.67	6.68–35.85	
BCS					0.006
2/5	6	3	50	18.76–81.24	
3/5	41	9	21.95	12.00–36.71	
4/5	8	3	37.50	13.68–69.43	
Vaccination status					0.847
vaccinated	25	6	24	11.50–43.43	
unvaccinated	30	9	30	16.66–47.88	
Clinical status					0.14
symptomatic	19	8	42.11	23.14–63.72	
asymptomatic	36	7	19.44	9.75–35.03	
County of origin					0.724
Alba	2	1	50	9.45–90.55	
Bistrița-Năsăud	1	0	0	0–79.35	
Cluj	49	14	28.57	17.85–42.41	
Satu-Mare	2	0	0	0–65.76	
Sălaj	1	0	0	0–79.35	
**Total**	**55**	**15**	**27.27**	**17.28–40.23**	

* Chi-square test; BCS—body condition score; CI—confidence interval.

**Table 3:** *E. cuniculi* prevalence based on nested PCR from fecal samples performed in 192 rabbits from the North-Western region of Romania.

Rabbit Data	No. Rabbits Tested	No. Positive Rabbits	Prevalence (%)	CI 95%	*p* *
Age					0.003
Young (≤4 months)	41	24	58.54	43.37–72.24	
Adults (>4 months)	151	48	31.79	24.89–39.59	
Sex					0.001
Male	81	25	30.86	21.86–41.60	
Female	104	40	38.46	29.68–48.06	
Unidentified	7	7	100	64.57–100	
Season of sampling					0.0002
spring	38	7	18.42	9.22–33.42	
summer	40	26	65	49.51–77.87	
autumn	75	28	37.33	27.26–48.65	
winter	39	11	28.21	16.54–43.78	
Rearing system					0.0001
Pet	40	2	5	1.38–16.50	
Family farm	152	70	46.05	38.32–53.98	
BCS					0.0003
2/5	9	1	11.11	1.99–43.50	
3/5	160	60	37.50	30.37–45.21	
4/5	23	11	47.83	29.24–67.04	
Vaccination status					0.823
vaccinated	90	35	38.89	29.47–49.22	
unvaccinated	102	37	36.27	27.60–45.95	
Clinical status					0.007
symptomatic	22	2	9.09	2.53–27.81	
asymptomatic	170	70	41.18	34.05–48.69	
County of origin					0.0001
Alba	36	20	55.56	39.58–70.46	
Bistrița-Năsăud	28	9	32.14	17.93–50.66	
Cluj	85	18	21.18	13.84–31.01	
Satu-Mare	28	23	82.14	64.41–92.12	
Sălaj	15	2	13.33	3.74–37.88	
**Total**	**192**	**72**	**37.50**	**30.96–44.53**	

* Chi-square test; BCS—body condition score; CI—confidence interval.

**Table 4:** *E. cuniculi* prevalence based on nested PCR from tissue samples performed in 52 rabbits from the North-Western region of Romania.

Rabbit Data	No. Rabbits Tested	No. Positive Rabbits	Prevalence (%)	CI 95%	*p* *
Age					0.975
Young (≤4 months)	13	10	76.92	49.74–91.82	
Adults (>4 months)	39	22	56.41	40.98–70.70	
Sex					0.004
Male	28	21	75.00	55.13–89.31	
Female	19	9	47.37	24.25–71.14	
Unidentified	5	2	40	5.27–85.34	
Season of sampling					0.004
spring	10	6	60	31.27–83.18	
summer	4	0	0	0–48.99	
autumn	16	10	62.50	35.43–84.80	
winter	22	16	72.73	49.78–89.27	
Rearing system					0.0002
Pet	17	9	52.94	30.96–73.83	
Family farm	32	22	68.75	51.43–82.05	
Wild	3	1	33.33	6.15–79.23	
BCS					0.707
2/5	3	1	33.33	6.15–79.23	
3/5	45	29	64.44	49.84–76.78	
4/5	2	1	50	9.45–90.55	
Undetermined	2	1	50	9.45–90.55	
Vaccination status					0.224
vaccinated	20	11	55.00	34.21–74.18	
unvaccinated	32	21	65.62	48.31–79.59	
Clinical status					0.0001
symptomatic	5	3	60.00	23.07–88.24	
asymptomatic	36	23	63.89	47.58–77.52	
undetermined	11	6	54.55	28.01–78.73	
County of origin					0.0001
Alba	5	3	60	23.07–88.24	
Cluj	44	28	63.64	48.87–76.22	
Satu-Mare	3	1	33.33	6.15–79.23	
**Total**	**52**	**32**	**61.54**	**47.02–74.70**	

* Chi-square test; BCS—body condition score; CI—confidence interval.

**Table 5:** *E. cuniculi* prevalence based on nested PCR from each type of tissue performed on 287 samples from 52 rabbits from the North-Western region of Romania.

Rabbit Tissue	No. Tissues Tested	No. Positive Tissues	Prevalence (%)	CI 95%	*p* *
Brain	52	11	21.15	12.24–34.03	0.48
Eye lens	51	7	13.73	6.81–25.72	
Kidney	45	12	26.67	14.60–41.94	
Urinary bladder	36	11	30.56	18.00–46.86	
Liver	29	9	31.03	17.28–49.23	
Lungs	28	4	14.29	4.03–32.67	
Heart	26	5	19.23	8.51–37.88	
Spleen	20	3	15	5.24–36.04	
**Total**	**287**	**63**	**21.95**	**17.55–27.09**	

* Chi-square test; CI—confidence interval.

**Table 6:** Comparison of *E. cuniculi* prevalence between ELISA and nested PCR performed on 212 rabbits.

Rabbit Data	No. of Rabbits	Frequency ELISA	Seroprevalence % (CI 95%)	Frequency Nested PCR	Prevalence % (CI 95%)	*p* *
Age						
Young (≤4 months)	51	13	25.49 (15.55–38.87)	31	60.78 (47.09–72.97)	0.0001
Adults (>4 months)	161	112	69.57 (62.07–76.15)	61	37.89 (30.76–45.58)	0.0001
Sex						
Male	92	50	54.35 (44.20–64.15)	36	39.13 (29.79–49.35)	0.039
Female	113	73	64.60 (55.44–72.81)	49	43.36 (34.59–52.57)	0.004
Unidentified	7	2	28.57 (8.22–64.11)	7	100 (64.57–100)	NA
Season of sampling						
spring	43	29	67.44 (52.52–79.51)	12	27.91 (16.75–42.69)	0.0001
summer	41	21	51.22 (36.48–65.75)	28	68.29 (53.02–80.44)	0.11
autumn	79	56	70.89 (60.09–79.75)	33	41.77 (31.53–52.78)	0.0001
winter	49	19	38.78 (26.43–52.75)	19	38.78 (26.43–52.75)	1.000
Rearing system						
Pet	40	22	55 (39.83–69.29)	10	25 (14.19–40.19)	0.004
Family farm	172	103	59.88 (52.42–66.92)	82	47.67 (40.34–55.11)	0.022
Vaccination status						
vaccinated	99	61	61.62 (51.77–70.59)	39	39.39 (30.34–49.24)	0.001
unvaccinated	113	64	56.64 (47.43–65.41)	53	46.90 (37.96–56.05)	0.14
Clinical status						
symptomatic	24	17	70.83 (50.83–85.09)	8	33.33 (17.97–53.29)	0.005
asymptomatic	188	108	57.45 (50.30–64.30)	84	44.68 (37.75–51.82)	0.012
**Total**	**212**	**125**	**58.96 (52.24–65.37)**	**92**	**43.40 (36.90–50.13)**	**0.001**

* Chi-square test; ELISA—enzyme-linked immunosorbent assay; CI—confidence interval; NA—not applicable.

**Table 7:** Comparison of *E. cuniculi* prevalence between qPCR and nested PCR performed on 31 rabbits.

Rabbit Data	No. of Rabbits	Frequency qPCR	Prevalence % (CI 95%)	Frequency Nested PCR	Prevalence % (CI 95%)	*p* *
Age						
Young (≤4 months)	2	2	100 (34.24–100)	1	50 (9.45–90.55)	NA
Adults (>4 months)	29	13	44.83 (28.41–62.45)	16	55.17 (37.55–71.59)	0.43
Sex						
Male	19	10	52.63 (31.71–72.67)	11	57.89 (36.28–76.86)	0.74
Female	10	4	40 (16.82–68.73)	5	50 (23.66–76.34)	0.65
Unidentified	2	1	50 (9.45–90.55)	1	50 (9.45–90.55)	NA
Season of sampling						
spring	3	2	66.67 (20.77–93.85)	2	66.67 (20.77–93.85)	NA
summer	8	2	25 (7.15–59.07)	1	12.50 (2.24–47.09)	0.51
autumn	9	6	66.67 (35.42–87.94)	6	66.67 (35.42–87.94)	NA
winter	11	5	45.45 (21.27–71.99)	8	72.73 (43.44–90.25)	0.16
Rearing system						
Pet	22	9	40.91 (23.26–61.27)	11	50 (30.72–69.28)	0.54
Family farm	9	6	66.67 (35.42–87.94)	6	66.67 (35.42–87.94)	NA
Vaccination status						
vaccinated	14	6	42.86 (21.38–67.41)	8	57.14 (32.59–78.62)	0.44
unvaccinated	17	9	52.94 (30.96–73.83)	9	52.94 (30.96–73.83)	NA
Clinical status						
symptomatic	10	6	60 (31.27–83.18)	5	50 (23.66–76.34)	0.65
asymptomatic	13	6	46.15 (23.21–70.86)	7	53.85 (29.14–76.79)	0.69
undetermined	8	3	37.50 (13.68–69.42)	5	62.50 (30.57–86.32)	0.30
**Total**	**31**	**15**	**48.39 (31.97–65.16)**	**17**	**54.84 (37.77–70.84)**	**0.61**

* Chi-square test; qPCR—quantitative polymerase chain reaction; CI—confidence interval; NA—not applicable.

**Table 8:** Diagnostic performance of the ELISA and qPCR using nested PCR as the reference standard.

	ELISA (%)	qPCR (%)
Sensitivity	58.7	58.8
Specificity	40.83	64.3
Positive Predictive Value	43.2	66.7
Negative Predictive Value	56.32	56.3
Accuracy	48.68	61.3
Kappa statistic (*p*-value *)	0 (0.94 *)	0.228 (0.19 *)
Prevalence nested PCR	43.39	54.83

* Chi-square test.

**Table 9:** Results of multivariate logistic regression analysis assessing risk factors associated with ELISA seropositivity (n = 351) and nested PCR positivity (n = 242) in rabbits.

Variable **		ELISA			nPCR	
	Odds Ratio	CI 95%	*p* *	Odds Ratio	CI 95%	*p* *
Age	4.4960	2.1902–9.2292	0.0001	0.5848	0.2921–1.1706	0.13
Sex	1.3473	0.8507–2.1336	0.204	1.0612	0.6106–1.8442	0.833
Season of sampling	0.5976	0.4703–0.7595	0.0001	1.1799	0.9095–1.5308	0.213
Summer	0.6829	0.3812–1.2029	0.187	2.7187	1.3922–5.4381	0.003
Autumn	1.1958	0.7785–1.8371	0.412	0.9312	0.5488–1.5757	0.79
Winter	0.4518	0.2596–0.7943	0.003	0.6796	0.3746–1.2182	0.194
Rearing system	0.7682	0.3450–1.7106	0.519	1.4574	0.6789–3.1288	0.334
Vaccination status	0.7509	0.4695–1.2009	0.232	0.7947	0.4544–1.3897	0.42
Clinical status	1.6674	0.5971–4.6564	0.329	0.7675	0.2635–2.2356	0.628

* Chi-square test; CI—confidence interval; Reference category **: Age ≤ 4 months; Sex = M; Season of sampling = Spring; Rearing system = Pet; Vaccination status = unvaccinated; Clinical status = asymptomatic.

**Table 10:** Rabbits that were tested with all methods of diagnosis for *E. cuniculi* from blood, urine, feces and tissues and their results (n = 6).

Rabbit No.	ELISA	nPCR			qPCR			Clinical Status
		Urine	Feces	Organs	Urine	Feces	Organs	
1	+	+	+	+	+	+	+	Neurological and renal signs over the course of 2 weeks.
2	+	+	−	+	+	−	+	Neurological signs over 1 month.
3	−	−	−	−	−	−	+	Neurological signs over 1 month.
4	−	−	−	−	+	−	+	Acute onset of seizures and death.
5	+	+	+	+	+	−	+	Head tilt to the left since youth.
6	−	−	+	+	−	−	+	Clinically healthy.

ELISA—enzyme-linked immunosorbent assay; qPCR—quantitative PCR; nPCR—nested PCR; +: positive; −: negative.

## Data Availability

The data presented in this study are available on request from the corresponding author due to owner privacy reasons.

## Abbreviations

The following abbreviations are used in this manuscript:

AIDS	Acquired Immunodeficiency Syndrome
BCS	Body Condition Score
CI	Confidence Interval
CIA	Carbon Immunoassay
CSF	Cerebrospinal Fluid
ELISA	Enzyme-linked Immunosorbent Assay
IFAT	Indirect Fluorescent Antibody Testing
IgG	Immunoglobulin G
IgM	Immunoglobulin M
PCR	Polymerase Chain Reaction
qPCR	Quantitative Polymerase Chain Reaction/Real-Time Polymerase Chain Reaction
RHD	Rabbit Hemorrhagic Disease
